# Clinical and biological significance of HAX-1 overexpression in nasopharyngeal carcinoma

**DOI:** 10.18632/oncotarget.7274

**Published:** 2016-02-09

**Authors:** Bo You, Xiaolei Cao, Xiaoyi Shao, Haosheng Ni, Si Shi, Ying Shan, Zhifeng Gu, Yiwen You

**Affiliations:** ^1^ Department of Otorhinolaryngology Head and Neck surgery, Affiliated Hospital of Nantong University, Nantong, Jiangsu Province, China; ^2^ Department of Pathology, Medical School of Nantong University, Nantong, Jiangsu Province, China; ^3^ Department of Pathogen and Immunology, Medical School of Nantong University, Nantong, Jiangsu Province, China; ^4^ Department of Rheumatology, Affiliated Hospital of Nantong University, Nantong, Jiangsu Province, China

**Keywords:** exosomes, HAX-1, nasopharyngeal carcinoma, angiogenesis, prognosis

## Abstract

HS1-associated protein X-1 (HAX-1) is an important marker in many types of cancers and contributes to cancer progression and metastasis. We examined the expression of HAX-1 in nasopharyngeal carcinoma (NPC) and experimentally manipulated its expression. We observed that HAX-1 expression is elevated in NPC and is correlated with lymph node metastasis, M classification, clinical stage, and poor prognosis. In addition, overexpression of HAX-1 promoted NPC proliferation both *in vitro* and *in vivo*. Exosomes are potential carriers of pro-tumorigenic factors that participate in oncogenesis. We found that NPC-derived exosomes are enriched in HAX-1 and accelerate NPC tumor growth and angiogenesis *in vitro* and *in vivo*. Furthermore, we demonstrated that oncogenic HAX-1 facilitates the growth of NPC when it is transferred via exosomes to recipient human umbilical vein endothelial cells (HUVECs). Oncogenic HAX-1 also increases the proliferation, migration, and angiogenic activity of HUVECs. Our findings provide unique insight into the pathogenesis of NPC and underscore the need to explore novel therapeutic targets such as HAX-1 to improve NPC treatment.

## INTRODUCTION

Nasopharyngeal carcinoma (NPC), a squamous epithelial cancer arising from the lateral wall surface of the nasopharynx, is the most common head and neck cancer, with an incidence of 30–80/100,000 each year in China [1, 2]. Local recurrence and distant metastasis are the major causes of death among NPC patients, because at the time of diagnosis a large number of patients have already reached an advanced stage and/or metastasis [3, 4]. Tumor angiogenesis is closely associated with the metastasis of NPC [5]. Thus, the identification of the underlying mechanisms associated with NPC angiogenesis, metastasis, and prognosis is crucial.

HS1-associated protein X-1 (HAX-1) is involved in a variety of important physiological processes including the regulation of apoptosis, cell motility, endocytosis, and interactions with the 3′untranslated regions (3′UTR) of a variety of proteins such as vimentin and DNA polymerase β [6–11]. While it is predominantly localized in the mitochondria, HAX-1 can also be found at endoplasmic reticulum and nuclear membrane [12]. HAX-1 expression is a predictor of tumorigenesis, growth, progression, invasion, and metastasis of many human malignancies [13, 14], and is overexpressed in many tumors [13, 15, 16] such as esophageal squamous cell carcinoma [17, 18], colorectal cancer [19], oral squamous cell carcinoma, lung cancer, lymphoma, melanoma [20], leukemia, myeloma, breast cancer, and hepatoma [21]. Recently, Li et al. reported that overexpression of HAX-1 is linked to poor prognosis in patients with esophageal squamous cell carcinoma [17]. HAX-1 regulates carcinoma cell migration and invasion via clathrin-mediated endocytosis of integrin αVβ6 [13]. However, the expression, clinical relevance, function, and mechanism of HAX-1 action in NPC has not previously been examined.

Exosomes, a population of membrane bound (30–100 nm in diameter) vesicles, are released from a diverse range of living cells, including tumor cells [22–25]. Interestingly, they influence the behavior of recipient cells by delivering complex biological information consisting of mRNAs, miRNAs, as well as soluble and transmembrane proteins. Exosomes directly stimulate the recipient cells by cell surface interactions, triggering downstream signaling events and transferring receptors from the cell of origin to the recipient cell [26–35]. Deciphering the specific molecular cargo of exosomes, and, in particular, tumor-derived exosomes, will help us to determine their functions. So far, tumor-derived exosomes have been found to suppress immune response, increase tumor progression, promote tumor invasiveness and metastasis, and confer multidrug resistance [36–39]. Tumor-derived exosomes are enriched in ΔNp73, which promotes oncogenic potential in acceptor cells and correlates with patient survival [40]. In addition, human nasopharyngeal carcinoma-derived exosomes promote tumor progression and T-cell dysfunction through the regulation of enriched exosomal microRNAs [41]. However, the expression of HAX-1 in NPC-derived exosomes and the relationship between exosomal HAX-1 and its functions has not been previously elucidated.

In this study we investigated the clinical and biological significance of exosomal HAX-1 in NPC. We first found that HAX-1 is overexpressed in NPC and is correlated with patients' clinicopathologic features and prognosis. Next, we observed that HAX-1 regulates the growth, apoptosis and migration, of NPC cells. Moreover, HAX-1 was selectively packaged in NPC-derived exosomes and conferred NPC growth by promoting angiogenesis.

## RESULTS

### HAX-1 overexpression in nasopharyngeal carcinoma has clinical significance

**Figure 1:**
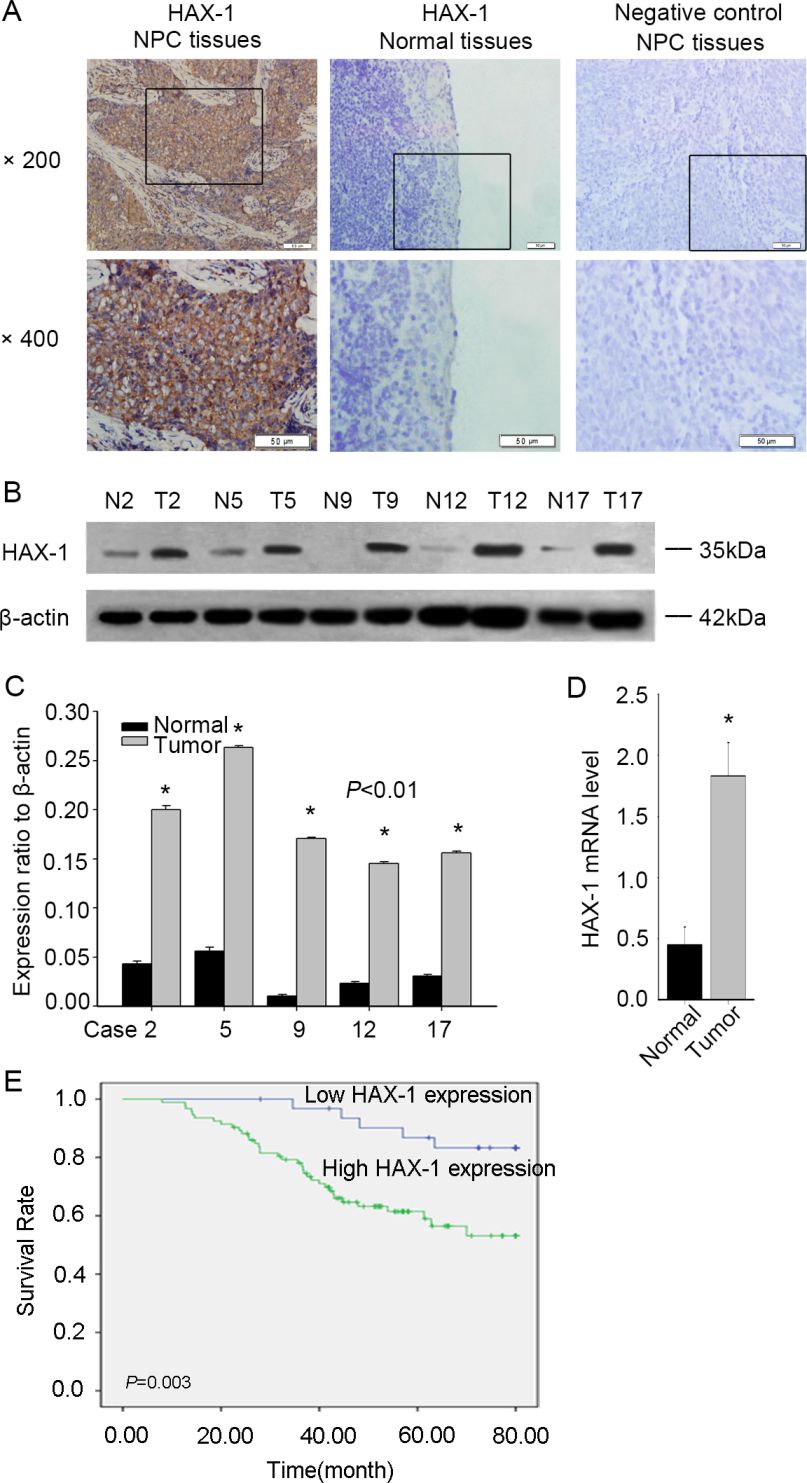
HAX-1 is highly expressed in NPC (**A**) Immunohistochemistry analysis of HAX-1 expression in NPC tissues. Scale bar, 50 μm. (**B**) Protein levels of HAX-1 in NPC (T) and nasopharyngeal tissues (N) by Western blotting. (**C**) Quantitative results of Western blot. (**D**) mRNA levels of HAX-1 in 20 paired NPC tissues by qPCR. (**E**) Kaplan–Meier survival curves of NPC patients based on HAX-1 expression status. *P < 0.05. β-actin was used as a loading control. The same experiment was repeated at least 3 times.

We used immunohistochemical analysis to detect the expression of HAX-1 in nasopharyngeal carcinoma (NPC) and non-cancerous nasopharyngeal tissues. The positive expression rate of HAX-1 in NPC (93/125; 74.40%) was higher than that in the non-tumor tissues (14/67; 20.90%) (Figure 1A, *P* < 0.01; Table 1). To confirm these findings, we examined the expression of HAX-1 in freshly obtained NPC tissues and non-tumor nasopharyngeal tissues. We again found markedly increased HAX-1 expression in NPC (Figure 1B–1D).

**Table 1:** Expression of HAX-1 in 125 human nasopharyngeal carcinoma tissues

Clinicopathological parameters	No. case	HAX-1 expression, *n*		*P*
		−∼+	++∼+++	
Gender				0.128
Male	99	22	77	
Female	26	10	16	
Age, yr				0.095
< 50	31	4	27	
≥ 50	94	28	66	
Smoking				0.819
No	92	23	69	
Yes	33	9	24	
T classification				0.472
T1–T2	96	23	73	
T3–T4	29	9	20	
N classification				0.000*
N0–N1	27	25	2	
N2–N3	98	7	91	
Distant metastasis				0.004*
No	100	31	69	
Yes	25	1	24	
TNM clinical stage				0.000*
I–II	21	20	1	
III–IV	104	12	92	

* Statistical analyses were performed by the Pearson *χ2* test. *P* < 0.05 was considered significant.

We next evaluated the relationship between clinicopathological characteristics and HAX-1 expression. As shown in Table 1, high HAX-1 expression was associated with lymph node metastasis (*P* < 0.01), M classification (*P* < 0.01), and clinical stage (*P* < 0.01). However, there was no association of HAX-1 expression with gender, age, smoking, or T classification (all *P* > 0.05).

Next, the prognostic significance of HAX-1 expression was assessed using Kaplan-Meier analysis. In 125 NPC cases, patients with HAX-1 overexpression had worse prognoses than those with negative expression (Figure 1E, *P* < 0.01). Univariate analyses showed that N classification (*P* < 0.01), M classification (*P* < 0.01), clinical stage (*P* < 0.01) and HAX-1 expression (*P* = 0.017) were correlated with a poor survival in NPC (Table 2). Multivariate analysis revealed that HAX-1 expression (*P* = 0.024, Table 3) was an independent prognostic factor in NPC patients.

**Table 2:** Survival status and clinicopathological parameters in 125 human nasopharyngeal carcinoma tissues

Clinicopathological parameters	Total	Survival status, *n*		*P*
		Alive	Dead	
Gender				1.000
Male	99	66	33	
Female	26	18	8	
Age, yr				0.826
< 50	31	20	11	
≥ 50	94	64	30	
Smoking				0.086
No	92	66	26	
Yes	33	18	15	
T classification				0.507
T1–T2	96	66	30	
T3–T4	29	18	18	
N classification				0.001*
N0–N1	27	25	2	
N2–N3	98	59	39	
Distant metastasis				0.000*
No	100	78	22	
Yes	25	6	19	
TNM clinical stage				0.002*
I–II	21	20	1	
III–IV	104	64	40	
HAX-1				0.017*
Low expression	32	27	5	
High expression	93	57	36	

* Statistical analyses were performed by the Pearson χ2 test. *P* < 0.05 was considered significant.

**Table 3:** Contribution of various potential prognostic factors to survival by cox regression analysis on 125 human nasopharyngeal carcinoma tissues

	Hazard ratio	*P*	95.0% Confidence interval
N classification			0.157–1.762
N1–N2 versus N3–N4	0.525	0.297	
Distant metastasis			0.409–2.231
M0 versus M1	0.955	0.915	
TNM clinical stage			0.518–3.794
I–II versus III–IV	1.401	0.507	
HAX-1 expression			0.105–0.856
Low versus High	0.300	0.024*	

Statistical analyses were performed by the Cox regression analysis.

* *P* < 0.05 was considered significant.

### HAX-1 promotes proliferation and migration, and reduces apoptosis of NPC cells

**Figure 2:**
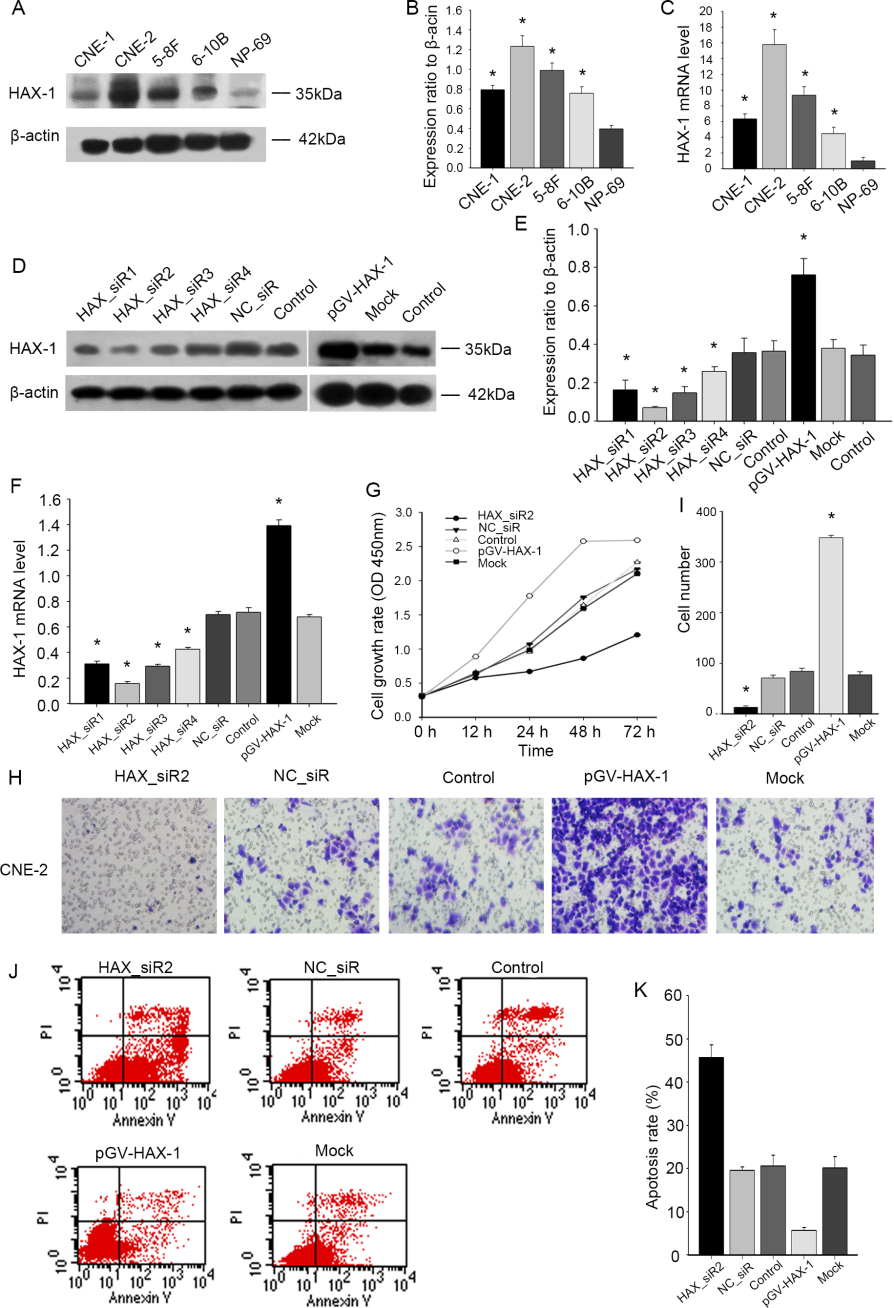
Effect of depletion or enforcing HAX-1 expression on proliferation, migration and apoptosis of NPC cells (**A**–**C**) Western blot and qPCR analysis of HAX-1 expression in CNE-1, CNE-2, 5-8F, 6-10B and NP-69. CNE-1, CNE-2, 5-8F, 6-10B are 4 kinds of human NPC cell lines and NP-69 is an immortalized normal nasopharyngeal epithelial cell line. (**D**–**F**) Interference efficiency was detected by western blot and PCR. (**G**) Cell proliferation was measured by CCK-8. (**H**–**I**) Cell migration analysis by transwell assays. (I) The number of cells that invaded through the membrane was counted in 10 fields under × 20 objective lens. *P* < 0.05. (**J**–**K**): Apoptosis rates were measured by FCM analysis after Annexin V/PI staining. K: Percentage of apoptotic cells. The data are mean ± SEM (*P* < 0.05, compared with the control). β-actin was used as a loading control. The same experiment was repeated at least three times.

To further investigate the potential biological roles of HAX-1 in NPC, we first evaluated the expression of HAX-1 in 4 human NPC cell lines and a normal nasopharyngeal epithelial cell line (NP69). As shown in Figure 2A–2C, the expression of HAX-1 in the 4 NPC cell lines was increased, especially in CNE-2 cells, as compared with NP69. Thus, CNE-2 cells were chosen for subsequent experiments.

To investigate the role of HAX-1 in NPC, CNE-2 cells were transfected with HAX-1-specific siRNA or a HAX-1 overexpression vector. As expected, HAX-1 was overexpressed in cells transduced by pGV-HAX-1, but was reduced in cells transduced by four different HAX_siRNAs with HAX_siR2 exhibiting the highest knockdown efficiency (Figure 2D–2F). To assess the effects of HAX-1 on CNE-2 cell proliferation, migration and apoptosis, we performed a cell-counting assay, transwell assay, and apoptosis analysis. Our results indicated that silencing HAX-1 expression decreased cell growth and migration and promoted apoptosis in NPC cells, while overexpression of HAX-1 promoted cell growth and migration and inhibited apoptosis (Figure 2G–2K).

### HAX-1 is necessary for tumor progression *in vivo*

**Figure 3:**
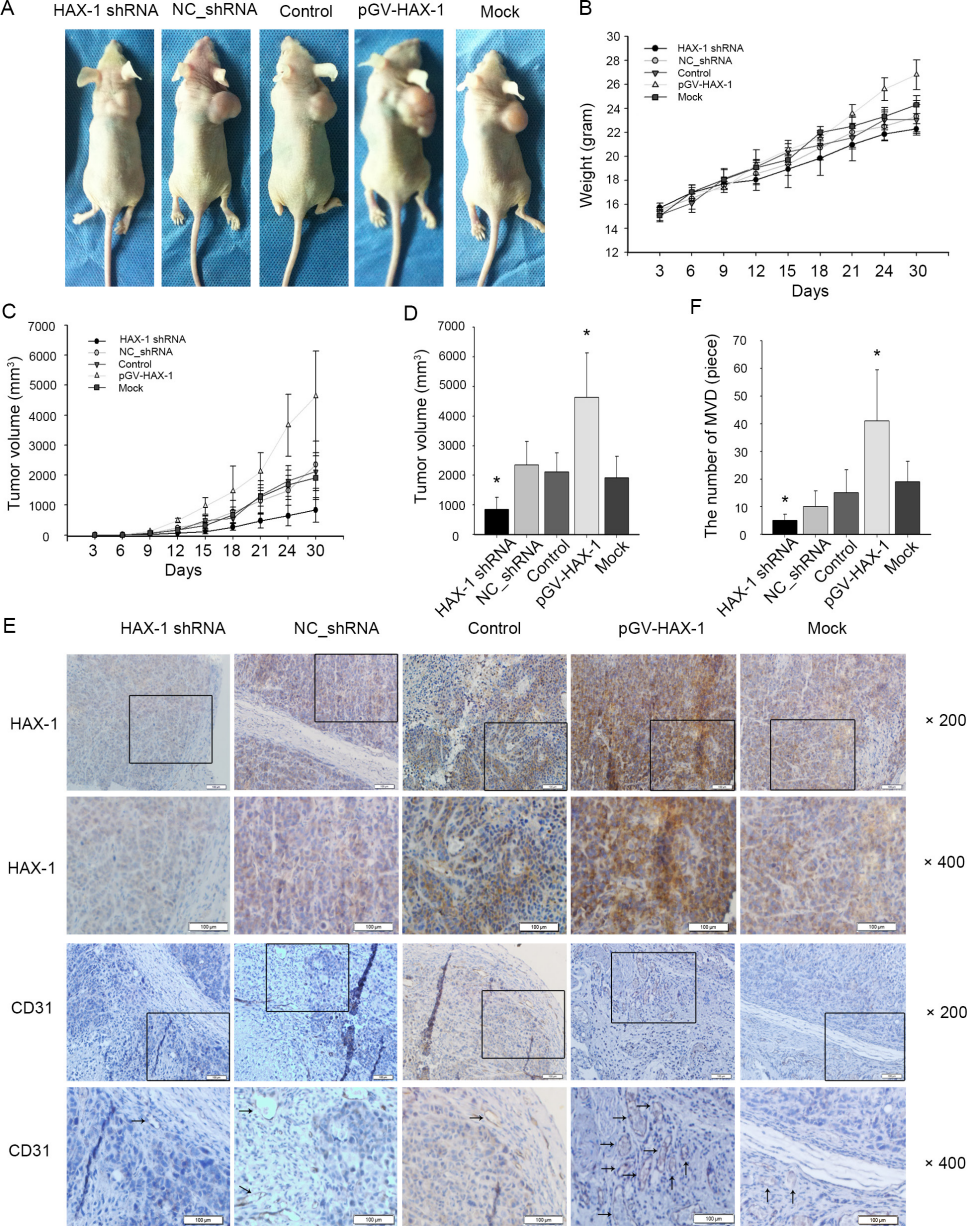
Constitutive silencing and activation of HAX-1 on growth of NPC in nude mice (**A**) Representative mice in HAX-1 silenced group, HAX-1 overexpressing group, and corresponding controls groups. (**B**) Body weights over time. (**C**) Tumor growth over time. (**D**) The 5 groups tumor volume at day 30. (**E**–**F**) Tumors were analyzed by immunohistochemistry for HAX-1 expression (E), and vascular density (E–F). Scale bar, 100 μm. *P < 0.05.

To confirm the effects of HAX-1 on tumor progression, we next investigated the function of HAX-1 *in vivo*. We established cancer cell lines stably overexpressing HAX-1 or stably transfected with lentiviral HAX-1 shRNA. Xenografts were established by subcutaneously injecting these different cell lines in nude mice, and tumor size was measured and recorded every three days. Growth curves showed reduced tumor volumes in xenografts with silenced HAX-1 expression. In contrast, overexpression of HAX-1 increased the growth of NPC tumors (Figure 3A–3D). The volumes and weights of transplanted tumors correlated with HAX-1 expression (Figure 3). These data provide evidence that HAX-1 promotes NPC growth *in vivo*. Interestingly, immunohistochemical analysis found that the HAX-1 overexpression groups showed more microvascular density (Figure 3E–3F).

### HAX-1 regulation of cell migration involves exosomes, which can be extracted and characterized from CNE-2 cells

**Figure 4:**
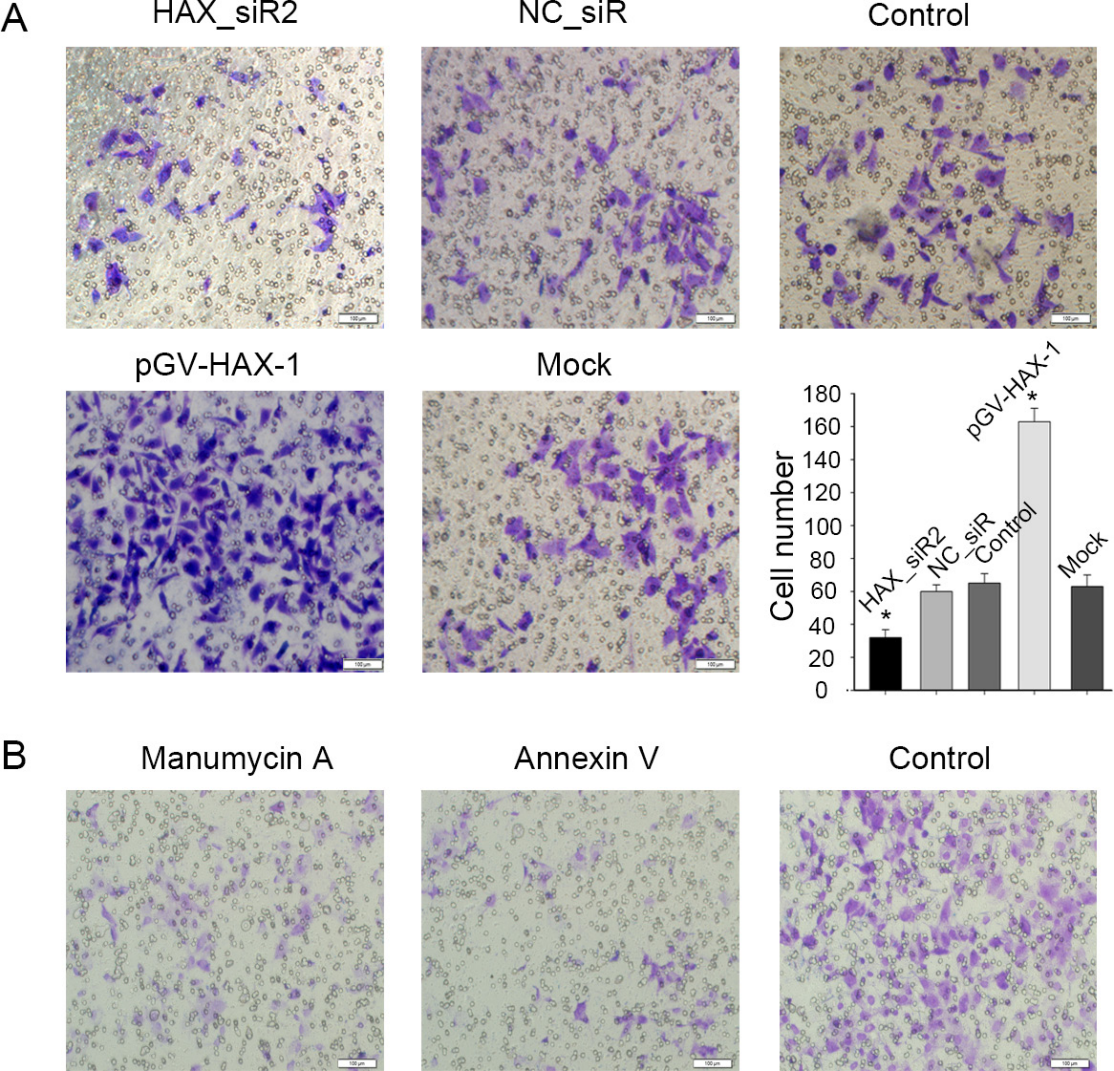
Exosomes might be the mechanism of HAX-1 regulation of NPC progression (**A**) HUVECs migration analysis by transwell assays. HUVECs were plated into the upper chambers, which were placed in the CM from CNE-2 cells. (**B**) HUVECs were plated into the upper chambers of a transwell system. 2.5 μMol/l manumycin A (neutral sphingomyelinase 2, nSMase2) or Annexin V derivatives (Diannexin) were added into CM from CNE-2 cells in the lower chambers.

To unravel the mechanisms by which HAX-1 regulates NPC progression, we examined the conditioned medium (CM) from CNE-2 cells. We plated human umbilical vein endothelial cells (HUVECs) into the upper chambers of a transwell chamber using the CM from CNE-2 cells. CM from CNE-2 cells with HAX-1 overexpression promoted the migration of HUVECs, while knockdown inhibited migration (Figure 4A). To conform the role of exosomes in the promotion of migration, we blocked the release and uptake of exosomes using 2.5 μMol/l manumycin A (neutral sphingomyelinase 2, nSMase2) and Annexin V derivatives (Diannexin). As shown in Figure 4B, blockade of exosome exchange reverses the effects of HAX-1 overexpression on migration. These results indicate that HAX-1 may regulate NPC progression through exosomes.

**Figure 5:**
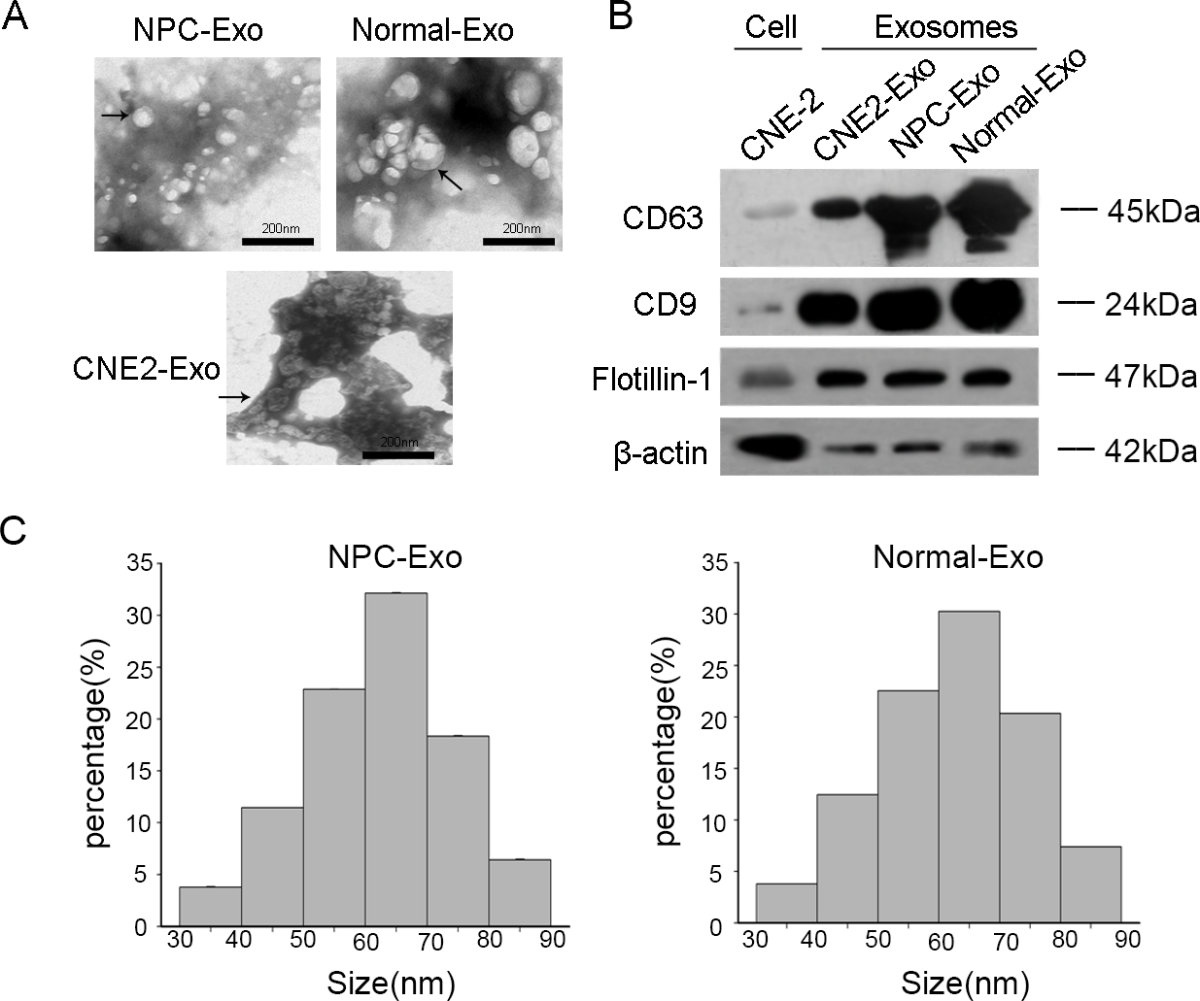
Characterization of exosomes (**A**) Representative electron microscopy image of exosomes. (**B**) Western blot analysis of CD63, CD9 and β-actin in exosomes and CNE-2 cells. Flotillin-1 was used as a loading control. (**C**) The number of exosomes was counted in 10 fields under TEM (transmission electron microscope).

We next isolated exosomes using ultracentrifugation. To ensure successful isolation, the collected exosomes were examined by TEM (transmission electron microscope). We confirmed a heterogeneous lipid bi-layer of vesicles approximately 30–100 nm in diameter, characterized as cup-shaped or irregular-shaped (Figure 5A). Western blot analysis showed isolated exosomes clusters were highly enriched in exosomal markers CD63 and CD9 and depleted of the cytoskeletal protein β-actin when compared with whole CNE-2 cell lysates (Figure 5B). No differences were observed in the total number of exosomes between NPC and normal cells (Figure 5C).

### NPC-derived exosomes accelerate NPC tumor growth and angiogenesis *in vivo*

**Figure 6:**
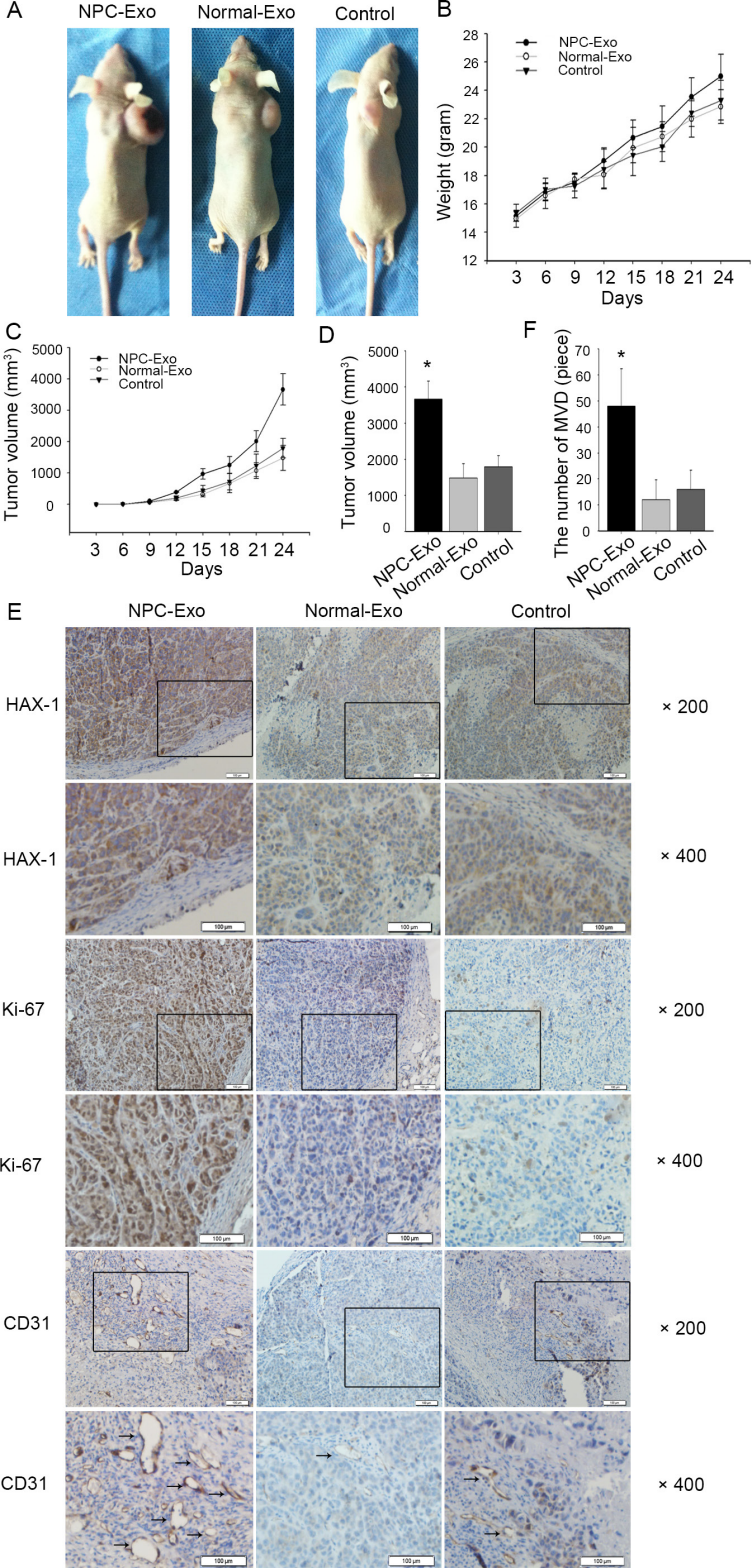
NPC-exosomes accelerate NPC xenograft growth and angiogenesis *in vivo* (**A**) Representative mice treated with NPC-exosomes, healthy donors-exosomes and PBS. (**B**) Body weight over time. (**C**) Tumor growth over time. (**D**) The 3 groups tumor volume at day 24. (**E**–**F**) Tumors were analyzed by immunohistochemistry for HAX-1 expression (E), proliferation (E), and vascular density (E–F). Scale bar, 100 μm. *P < 0.05.

Tumor-derived exosomes play an important role in tumor progression and metastasis by acting as intercellular communicators [37, 40]. Here, we examined the effects of NPC-exosomes on NPC tumorigenicity *in vivo*. A mouse NPC xenograft model was established and NPC-exosomes were injected three times a week for 3 weeks. Throughout the phase and at the end point, we observed that tumor sizes in those mice injected with NPC-exosomes were larger than in controls (injected with exosomes from healthy donors, Figure 6A–6D). Accordingly, these tumors had a higher number of cells that were positive for the proliferative marker Ki-67 than controls (Figure 6E). Moreover, mice injected with NPC-exosomes had higher levels of HAX-1 in the xenograft tumor than mice injected with exosomes from healthy donors (Figure 6E). Interestingly, immunohistochemistry suggested that tumors grown in the presence of NPC-exosomes exhibited enhanced vascularization compared with controls (Figure 6E–6F).

**Figure 7:**
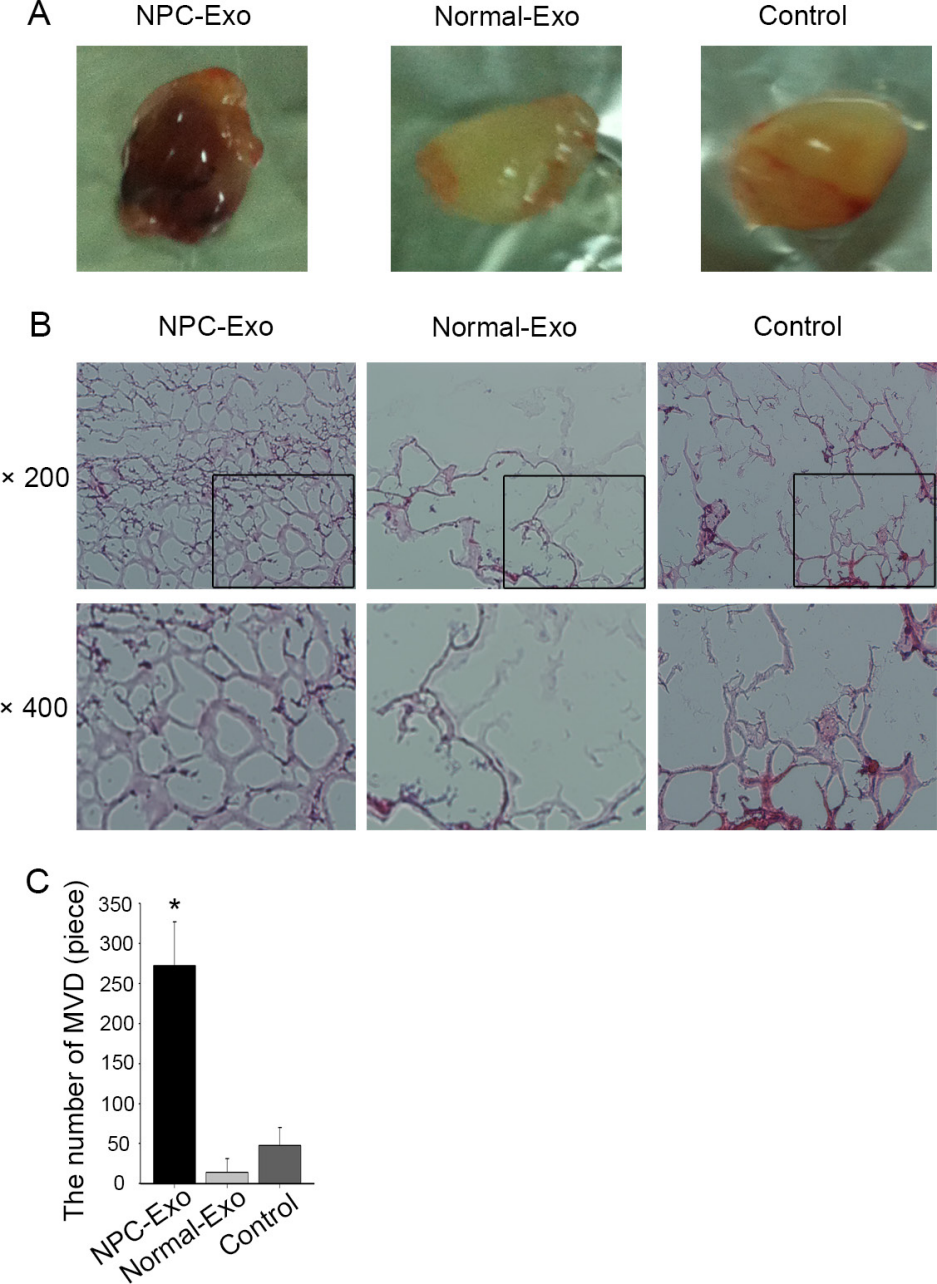
*In vivo* proangiogenic effects of NPC-exosomes Matrigel in the presence or absence of NPC-exosomes was injected subcutaneously into mice. After 7 days, Matrigel were recovered and stained with hematoxylin and eosin. (**A**) Gross observation, NPC-exosomes significantly promoted angiogenesis. (**B**) Representative micrographs of hematoxylin and eosin staining of Matrigel. (**C**) Quantitative evalu ation of angiogenesis. Angiogenesis was evaluated as the percentage of vessel area, and data are expressed as mean ± SEM (*P < 0.05, compared with the control). The same experiment was repeated at least three times.

To further evaluate the proangiogenic activity of NPC-exosomes, NPC-exosomes within Matrigel were injected subcutaneously into mice. As shown in Figure 7, a massive formation of vessel-like structures was observed with HE staining. In contrast, no apparent vessel formation was detected in the Matrigel with exosomes from healthy donors and without exosomes. These data indicate that NPC-exosomes have angiogenic activities.

### NPC-exosomes are enriched in HAX-1 and modulate proliferation, migration and angiogenesis in HUVECs

**Figure 8:**
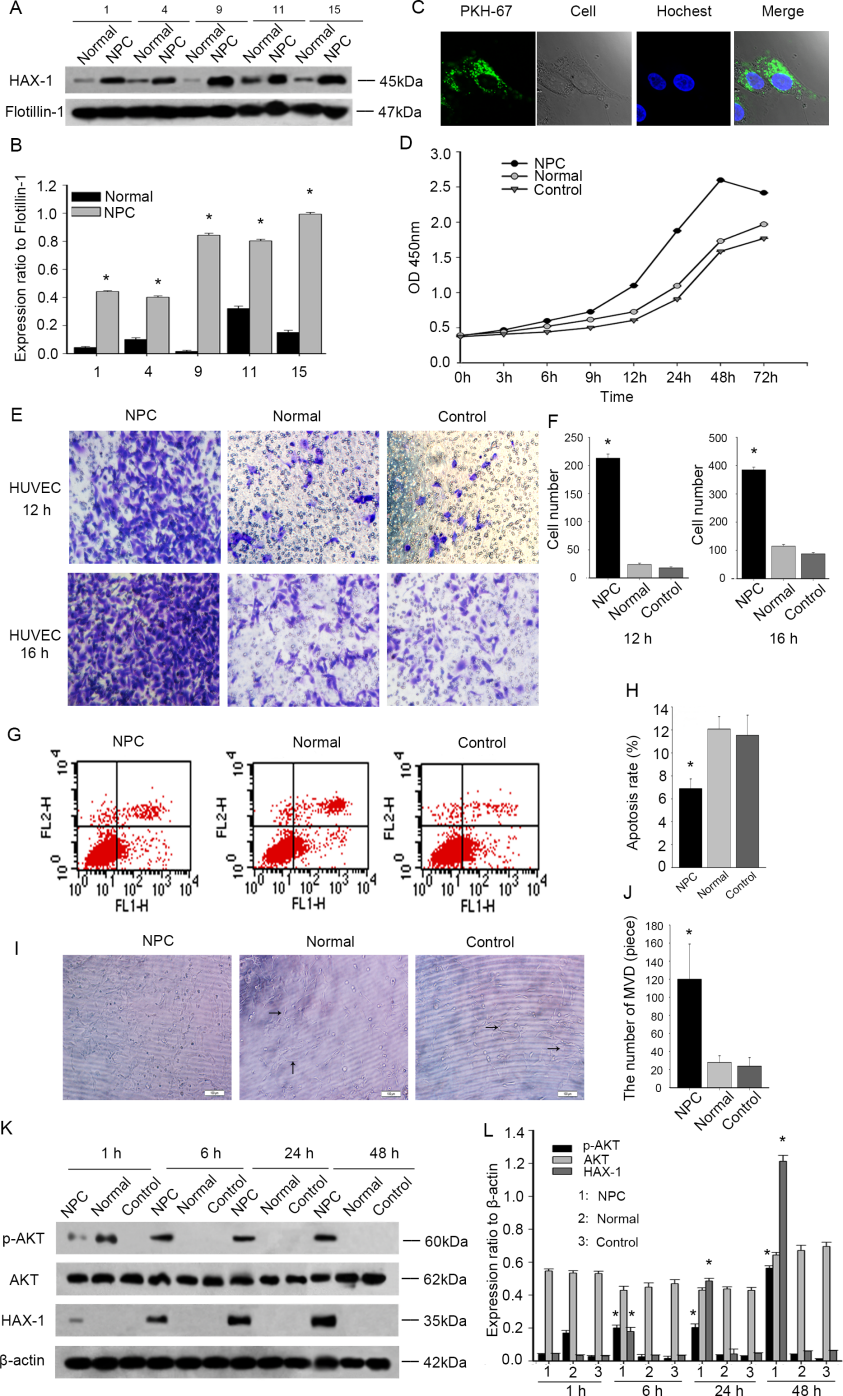
NPC-exosomes are enriched in HAX-1 which increases proliferation, migration and angiogenesis in HUVECs (**A**–**B**) Western blot analysis of HAX-1 expression in exosomes isolated from NPC and normal people. Flotillin-1 was used as a loading control. (**C**) Uptake of PKH67-labeled exosomes by HUVECs. In the confocal microscopy image, Hoechst staining was used to detect nuclei of cells (blue) and PKH67 was used to label the exosomes (green). (**D**) Proliferation analysis was performed after exosome addition to HUVECs. (**E**–**F**) Cell migration analysis by transwell assays. Each time point was derived from three independent experiments. (**G**–**H**) Apoptosis rates were measured by FCM analysis after Annexin V/PI staining. (**I**–**J**) Representative micrographs of capillary-like structure formation on Matrigel by HUVECs unstimulated (Control) and stimulated with exosomes isolated from NPC and normal people. *P < 0.05. (**K**–**L**) Western blot of P-AKT, AKT and HAX-1 in HUVECs incubation with exosomes isolated from NPC and normal people. β-actin was used as a loading control.

Particular populations of proteins are selectively packaged in exosomes and transferred in a cell type-specific fashion [40]. We found that HAX-1 is enriched in exosomes from NPC patients when compared with exosomes from healthy donors (Figure 8A–8B). NPC-exosomes labeled with PKH67 dye were internalized by HUVECs after a 30-minute co-incubation at 37°C (Figure 8C). The recipient HUVECs showed a time-dependent upregulation of HAX-1 after incubation with NPC-exosomes (Figure 8K–8L). These data support the idea that HAX-1 is transferred via exosomes in a cell type-specific manner. We next investigated the proangiogenic activity of NPC-exosomes on HUVECs *in vitro*. CCK8 assays revealed that NPC-exosomes (200 ug/ml protein) stimulated the proliferation of HUVECs when compared with control exosomes (Figure 8D). Furthermore, NPC-exosomes increased the number of migrated HUVECs in a time-dependent manner (Figure 8E–8F). Apoptosis analysis showed that NPC-exosomes inhibited the apoptosis of HUVECs (Figure 8G–8H). Moreover, stimulation of HUVECs with NPC-exosomes promoted their formation of capillary-like structures on Matrigel after incubation at 37°C for 6 hours (Figure 8I–8J). After stimulation with NPC-exosomes, HUVECs also had enhanced expression of p-AKT in a time-dependent manner (Figure 8K–8L). These data demonstrate that NPC-exosomes facilitate cell proliferation, migration, and angiogenesis and inhibit the apoptosis of HUVECs by transferring HAX-1 via exosomes to HUVECs and activating p-AKT.

### Exosomal HAX-1 modulates proliferation, migration and angiogenesis in HUVECs

**Figure 9:**
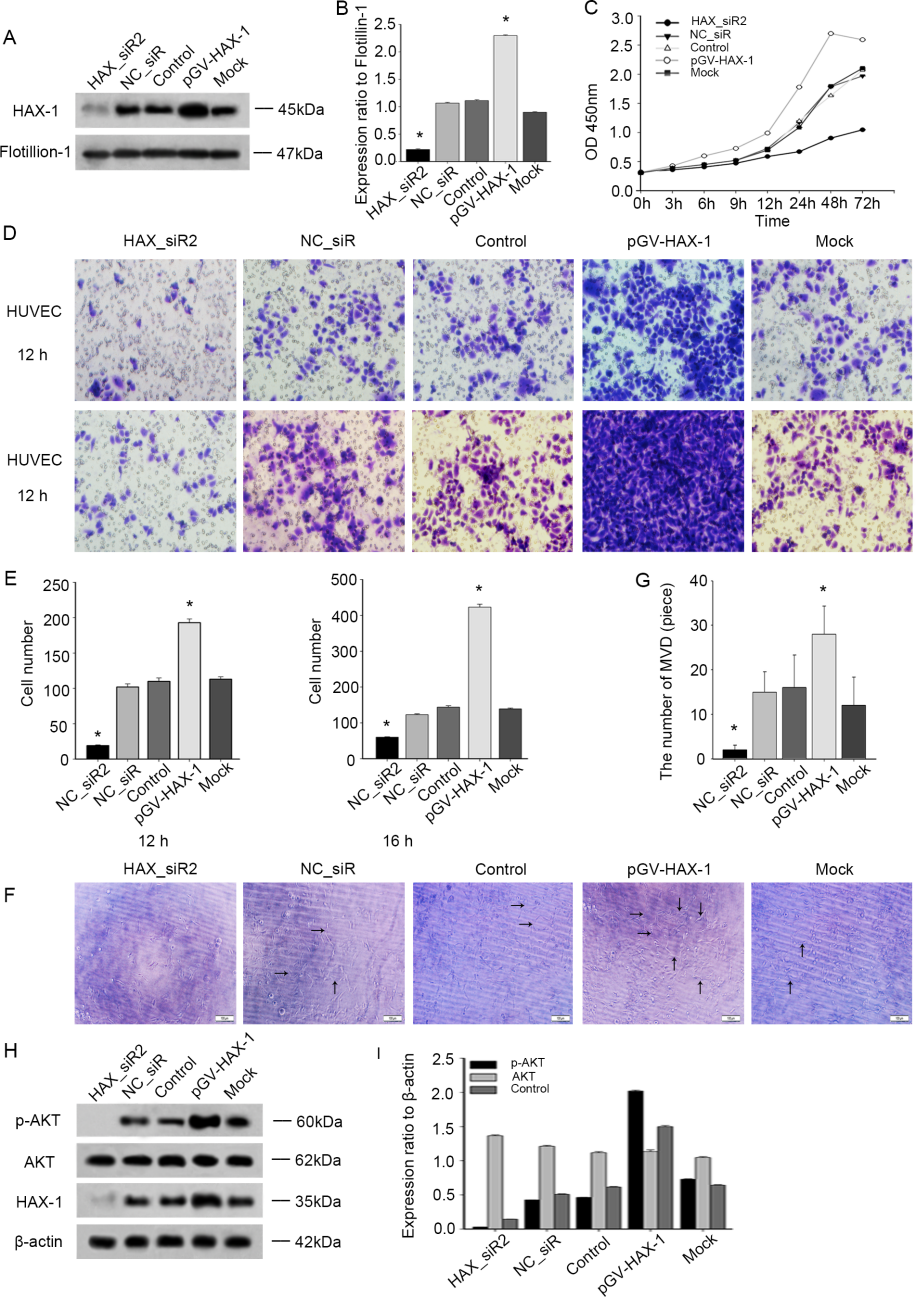
Transfer of exosomal HAX-1 increases proliferation, migration and angiogenesis effects in HUVECs (**A**–**B**) Western blot analysis of HAX-1 expression in exosomes isolated from culture media of HAX_siR2, NC_siR, control, pGV-HAX-1 and mock CNE-2 cells. Flotillin-1 was used as a loading control. (**C**) HUVECs proliferation was measured after treating with exosomes containing different levels of HAX-1 protein. (**D**–**E**) Cell migration analysis by transwell assays. (**F**–**G**) Representative micrographs of capillary-like structure formation on Matrigel. *P < 0.05. (**H**–**I**) Western blot of P-AKT, AKT and HAX-1 in HUVECs incubation with exosomes. β-actin was used as a loading control. All the same experiment was repeated at least 3 times.

To evaluate whether exosomal HAX-1 was critical for modulating exosomal induction of proliferation, migration and angiogenesis, HUVECs were stimulated with exosomes with different levels of HAX-1 protein. Exosomes were isolated from the culture media of CNE-2 cells transfected with HAX_siR2 or pGV-HAX and analyzed. Western blot confirmed that these exosomes contained different levels of HAX-1 protein (Figure 9A–9B). As expected, exosomal HAX-1 drastically increased the proliferation, migration and angiogenesis of HUVECs (Figure 9C–9G). Moreover, HAX-1 was transferred via exosomes to recipient HUVECs and intracellular downstream pathways were activated (Figure 9H–9I).

### Exosomal HAX-1 accelerates NPC xenograft tumor growth and angiogenesis

**Figure 10:**
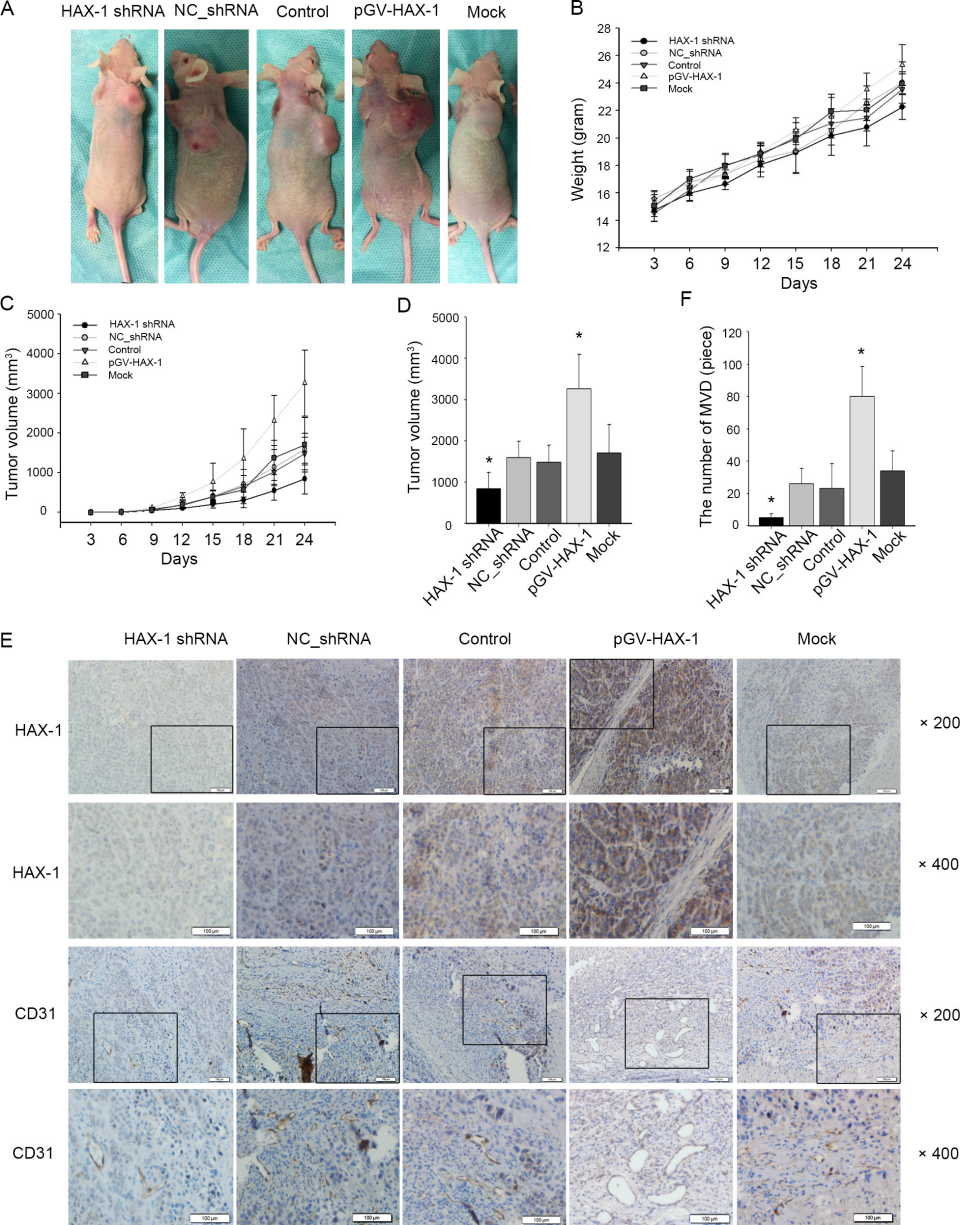
Exosomal HAX-1 accelerate NPC xenograft tumors growth and angiogenesis (**A**) Representative examples of mice treated with exosomes with different levels of HAX-1 protein. (**B**) Body weights over time. (**C**) Tumor growth over time. (**D**): The 5 groups tumor volume at day 24. (**E**–**F**) Tumors were analyzed by immunohistochemistry for HAX-1 expression (E) and vascular density (E–F). Scale bar, 100 μm. *P < 0.05.

To investigate whether the above findings hold true *in vivo*, we next injected exosomes with different levels of HAX-1 protein into an NPC xenograft mouse model. In agreement with our data above, exosomes with high level of HAX-1 protein accelerated tumor growth (Figure 10A–10D). Moreover, mice injected with high levels of exosomal HAX-1 protein had upregulated HAX-1 expression and vascularization (Figure 10E–10F). Taken together, these data demonstrate that HAX-1 facilitates the growth of NPC by transferring HAX-1 via exosomes to recipient HUVECs and increasing the proliferation, migration, and angiogenesis, while inhibiting apoptosis.

## DISCUSSION

Carcinogenesis and development of NPC are complicated biological processes characterized by uncontrolled proliferation and migration. Thus, it is crucial to identify mechanisms associated with NPC progression, metastasis, and prognosis to facilitate an early diagnosis, support prognosis prediction, and to develop novel therapeutic strategies. In the present study, we investigated the influences of HAX-1 on NPC. Our findings suggest that overexpressed HAX-1 promotes the growth of NPC via exosomes to recipient HUVECs and increases proliferation, migration and angiogenesis.

HAX-1 was identified more than 10 years ago as a novel protein with ubiquitous tissue expression [42]. HAX-1 binds with many cellular and viral proteins suggesting its involvement in multiple signaling pathways and cellular processes. HAX-1 has also been shown to bind to the 3′untranslated regions of certain mRNAs and could therefore contribute to the regulation of transport and/or stability of such transcripts [9]. HAX-1 is an important target of study in the field of cancer research because of its involvement in the regulation of apoptosis and cell migration, key processes in carcinogenesis and metastasis. HAX-1 is an inhibitor of apoptosis and interacts directly with some apoptosis-regulating proteins. HAX-1 is also a link between cytoskeleton polycystic kidney disease protein (PKD2) and the actin cytoskeleton [43] and forms a quaternary complex with cortactin, Rac and Gα13, a protein that is primarily involved in the regulation of cell migration [14]. HAX-1 regulates carcinoma cell migration and invasion by interacting with the β6 subunit and regulates clathrin-mediated endocytosis of αvβ6 integrins [13]. These data support the role of HAX-1 as a regulator of cell migration. However, the role of HAX-1 in NPC carcinogenesis has remained largely undetermined.

The present study is the first to find that HAX-1 is upregulated in NPC. This finding is consistent with previous reports from other tumors [13, 15, 16]. Overexpressed HAX-1 is associated with lymph node metastasis, distant metastasis, and clinical stage (all *P* < 0.05). Furthermore, survival analysis confirmed that NPC patients with HAX-1 overexpression have a shorter survival time. In addition, univariate and multivariate analyses revealed that overexpressed HAX-1 was an independent predictor of poor prognosis. These results show that HAX-1 functions as a potential oncogene with an important role in the progression and migration of NPC and is a novel prognostic marker for NPC patients. Therefore, to further determine the biological functions of HAX-1 in NPC, we knocked down or increased HAX-1 expression in CNE-2 cells. We found that HAX-1 promotes the growth and migration of NPC cells and inhibits apoptosis both *in vitro* and *in vivo*.

To unravel the mechanisms by which HAX-1 regulates NPC progression, we focused on exosomes. Exosomes could influence the phenotypes of recipient cells by delivering complex biological information between cells [44]. Given that the protein composition of extracellular vesicles is similar to the parental cell type; tumor-derived exosomes can contain tumor-specific antigens as well as oncoproteins and mRNAs from the tumor cells. For example, tumor-derived circulating exosomes are enriched in ΔNp73, an exosomal miRNA which has prognostic value in advanced disease stage in colon cancer, breast cancer, and lung cancer [40, 45–47]. In our experiments, we observed that exosomes from NPC patients contained higher levels of HAX-1 protein than exosomes from healthy donors. Likewise, HAX-1 levels were higher in exosomes coming from cells overexpressing HAX-1 but absent in exosomes coming from cells with knockdown of HAX-1 expression. Both approaches suggest that those cells expressing higher levels of HAX-1 could package more HAX-1 in exosomes. Previous studies have shown that tumor-derived exosomes play an important role in tumor progression and metastasis by acting as intercellular communicators [37, 40]. For example, bladder cancer cell lines shed exosomes containing proteins important for tumor progression, and these exosomes inhibit tumor cell apoptosis through AKT and ERK pathways [48]. Here, we examined the effects of NPC-exosomes on NPC tumorigenicity. Our data find that NPC-derived exosomes accelerate NPC tumor growth and angiogenesis. In addition, the effects of NPC-exosomes depend on exosomal HAX-1.

The cargo of exosomes is particularly interesting as exosomes excreted from one cell are known to be able to fuse with surrounding cells, and thus have the potential to initiate signaling responses [26]. This is of particular relevance in tumorigenesis. Our data find that NPC-derived exosomal HAX-1 can alter the phenotype of HUVECs after internalization of exosomes. Once incorporated, HAX-1 seems functionally active, as there is a significant increase in its downstream effects by up-regulation of the AKT pathways. Undeniably, additional novel, specific oncogenic roles for HAX-1 will help us to clarify its functions when encapsulated and released into the environment by the tumor.

In summary, our data offer convincing evidence that HAX-1 is overexpressed in NPC and that the level of HAX-1 is associated with clinical progression and poor prognosis. Overexpressed HAX-1 promotes NPC cells growth and migration and decreases apoptosis. Furthermore, we demonstrate that HAX-1 facilitates the growth of NPC by its transferring via exosomes to recipient HUVECs, thereby increasing proliferation, migration, and angiogenesis. Our findings provide unique insights into the pathogenesis of NPC and underscore the need to explore novel therapeutic targets for NPC treatment.

## MATERIALS AND METHODS

### Tissue specimens and Ethics statement

All tissues were obtained from NPC patients at the Otorhinolaryngology Head and Neck surgery, Affiliated Hospital of Nantong University, China. 67 non-cancerous nasopharyngeal samples were collected from patients with clinical symptoms suggestive of NPC, but ruled out by biopsy. The patients had not received any therapy prior to biopsy. All participants gave consent to the study, which was approved by the Ethics Committee of the Affiliated Hospital of Nantong University.

### Immunohistochemical staining

Immunohistochemistry was carried out according to previous reports [49]. For assessment of HAX-1, the staining intensity and relative percentage of immunostained cells were analyzed and evaluated by two pathologists blind to disease status. The intensity of each section was assessed as strong (3), moderate (2), weak (1), or negative (0), as well as semi-quantitatively using the following scale: ≤ 5% of cells (0), 6–25% of cells (1), 26–50% of cells (2), 51–75% of cells (3), ≥ 75% of cells (4). The scoring results of intensity and extent were multiplied to give a composite score ranging from 1 to 12 for each section: 0, –(negtive); 1–4, + (weak positive); 5–8, ++ (moderate positive); 9–12, +++ (strong positive). When evaluating Ki-67 expression, 51–100% of positively stained cell nuclei were in the high expression group and 0–50% in the low expression group.

### Microvasculature density counting

Microvasculature marked by CD31 was counted. CD31 positive cell clusters forming lumen or vessels were counted as individual microvessels [50]. Unstained lumen were considered artifacts, even if they contained blood or tumor cells. Sections of nude mice xenografts were scanned under light microscopy at low power (×20 objective and ×10 ocular) to identify vascular intensive areas. Individual microvessels were then counted at high power (×40 objective and ×10 ocular) in five fields and the mean vessel count was used as the microvasculature density (MVD).

### Western blot analysis

For western blot analysis, we used the following antibodies: anti–HAX-1 (1:500, Santa Cruz Biotechnology, CA, USA), anti–p-AKT (1:1000, Santa Cruz Biotechnology), anti–AKT (1:1000, Santa Cruz Biotechnology), anti–Flotillin-1 (1:1000, Santa Cruz Biotechnology), anti–CD63 (1:1000, Sangon Biotech, Shanghai, China), and anti–CD9 (1:1000, Sangon Biotech, Shanghai, China), as well as anti-β-actin (1:2000, Santa Cruz Biotechnology) as a loading control.

### Quantitative real-time PCR

Quantitative real-time PCR were carried out as previously described [49]. The primers used for quantitative real-time PCR were purchased from Biomics Biotechnologies Co., Ltd (Nantong, China) and were as follows: HAX-1 forward: 5′-TCAATAGCATCTTCAGCGATATG-3′ reverse: 5′-GTCCCTCCCGTAGTCTCTC-3′. The expression of each gene was normalized by expression of GAPDH.

### Cell cultures

All the cell lines used in this research were purchased from the Wuhan Institute of Cell Biology, China Center for Type Culture Collection. 4 kinds of human NPC cell lines CNE-1 (high differentiation), CNE-2 (low differentiation), 5–8F (high tumorigenesis and metastasis) and 6–10B (low tumorigenesis and metastasis) maintained in RPMI 1640 (GibCo BRL, Grand Island, NY) supplemented with 10% fetal bovine serum (FBS, GibCo). The immortalized normal nasopharyngeal epithelial cell line NP69 cultured in Keratinocyte-SFM (Invitrogen, Carlsbad, CA). HUVEC cells (human umbilical vein endothelial cells) were maintained in DMEM with low glucose (DMEM-LG, Gibco) supplemented with 10% fetal bovine serum (FBS, GibCo). Each cell line was maintained at 37°C in a humidified atmosphere and 5% CO_2_ according the recommendations of the providers.

### Immunofluorescence microscopy

Cells were fixed with 4% paraformaldehyde and blocked with 1% normal donkey serum. Cells were then incubated with primary antibodies overnight. Staining was visualized using anti-rabbit or anti-mouse IgG Alexa Flour 488 (Molecular Probes), counterstained with Hoechst and observed with a fluorescence microscope.

### Plasmids and siRNAs

The negative control siRNA (NC_siR) and specific siRNAs targeting HAX-1 (Accession No. NM_006118 form NCBI GenBank) were designed and obtained from Biomics Biotechnologies Co. Ltd (Nantong, China), and the BLAST analysis of siRNA sequences was carried out to ensure on homology with others genes in human. The sequences of siRNAs are shown in Table 4. pcDNA-SEQR-based HAX-1 reporter plasmid was purchased from genechem (Shanghai, China) (GOSE44428). The mock GV141 vector was used as a negative control. The lentiviral packaging HAX-1 shRNA was designed and obtained from genechem (Shanghai, China).

**Table 4:** Sequences of siRNA targeting HAX-1

siRNA names	Sequences(5′–3′)
HAX_siR 1	Sense: GGAUACGUUUCCACGAUAAdTdT Antisense: UUAUCGUGGAAACGUAUCCdTdT
HAX_siR 2	Sense: GAGUGAUGCAAGAAGUGAAdTdT Antisense: UUCACUUCUUGCAUCACUCdTdT
HAX_siR 3	Sense: GAAGCAGAUAGCAGUCCUAdTdT Antisense: UAGGACUGCUAUCUGCUUCdTdT
HAX_siR 4	Sense: GGACUGUGGUGGACAGUGAdTdT Antisense: UCACUGUCCACCACAGUCCdTdT
NC_siR	Sense: UUCUCCGAACGUGUCACGUdTdT Antisense: ACGUGACACGUUCGGAGAAdTdT

### Transfections and transductions

Cells were grown in six-well plates and transfected with 1 μg of DNA plasmids or 20 pmol siRNA using Lipofectamine 2000 (Invitrogen) following the recommendations of the manufacturer. Transfected cells and culture medium were used for the subsequent experiments 48 h or 96 h after transfection.

### Cell viability assay

Cells were seeded in 96-well plates (20, 000 cells/well) and incubated for 24 h. 10 μl per well of CCK-8 Kit reagents were added and incubated for 2 h incubation at 37°C, and the absorbance was read at 450 nm in an automated plate reader.

### Transwell assays

For the migration assay, cells were trypsinized and seeded in medium without serum; then 1 × 10^5^ cells were plated into the upper chambers of cell culture inserts (24-well type, 8-um pore size, Corning, NY, USA), which were placed in medium containing 10% fetal bovine serum with or without exosomes. After 12 or 16 h of incubation, the cells attached to the upper side of the filter were mechanically removed, and the cells that had migrated to the undersurface of the membrane were fixed and stained with crystal violet. Digital images were obtained from the membranes, and ten random fields were counted.

### Cell apoptosis assay

1 × 10^6^ cells were harvested and washed with PBS. The cells were resuspended in Binding Buffer, followed incubated with Annexin V-FITC and PI for 15 min at room temperature. Cells were then analyzed by flow cytometry (FCM) analysis using BD CELLQuest software (BD Biosciences, USA).

### Animal xenograft tumor model

BALB/c athymic nude mice (4 to 6 weeks old) were purchased from Shanghai Laboratory Animal Center, China and kept in a specific pathogen-free environment. All mouse experiments followed institutional guidelines and were approved by the committee on the Ethics of Animal Experiments of Nantong University. Mice received 1 × 10^6^ tumor cells subcutaneously. For exosome treatment, 1 day after cell inoculation, 20 ug of total exosome protein (in a total volume of 200 ul of PBS) was injected by tail vein three times a week for 3 weeks. The date at which the first grossly visible tumor appeared was recorded, and the tumor growth, body weight and signs of any sickness was monitored daily with sliding calipers. All the mice were sacrificed 30 days after tumor inoculation. Half of the primary tumors were fixed in 10% formalin overnight and subjected to routine histological examination by investigators who were blinded to the tumor status. The other half was frozen at −80°C for later research.

### Recovery of human blood serum

Venous blood from healthy volunteers and NPC patients was collected on isocitrate anticoagulant solution and centrifuged at 300 g for 5 min. The resulting platelet-rich plasma was subjected to a second centrifugation at 3,000 g for 20 min to remove cell debris, and then processed for exosome purification.

### Exosome isolation and purification

Exosomes were obtained as previously described [40, 51, 52]. Exosomes were collected by differential centrifugation and centrifuged at 300 × g for 5 minutes, 3,000 × g for 20 minutes, 6,000 × g for 40 minutes, 10,000 × g for 60 minutes, cell-free supernatants were ultracentrifuged at 100,000 × g for 1 h at 4°C (Beckman 90 Ti rotor). Exosomes were washed once with exosome-depleted PBS and submitted to a second ultracentrifugation in the same conditions. To quantify the protein content, exosomes pellets were suspended in exosome-depleted PBS and estimated by a BCA protein assay kit (PIERCE, Rockford, IL, USA) and exosomes were stored at −80°C and used within 5 days after isolation.

### Transmission electron microscopy

Exosomes were fixed with 2.5% glutaraldehyde in PBS for 2 h. After exosomes were washed, they were ultracentrifuged and suspended in 100 ul PBS. A 20 ul drop of exosomes was loaded onto a formvar/carbon-coated grid, negatively stained with 3% aqueous phosphotungstic acid for 1 minute and observed by transmission electron microscope (TEM, JEM-1230, JEOL, Tokyo, Japan).

### Exosome labeling

For the exosome-tracking experiments, purified exosomes were fluorescently labeled using PKH67 membrane dye (Sigma-Aldrich) according to the manufacturer's protocol. Briefly, labeled exosomes were washed with DMEM, collected by ultracentrifugation as described above and resuspended in DMEM. HUVEC cells were seeded and incubated for 6 h with 200 ug/ml of PKH67-labeled exosomes. After that, the cells were subsequently fixed with 4% paraformaldehyde and washed twice with PBS. Nuclei were stained with Hoechst for 15 min and the sections were mounted with PBS glycerol. Images were collected with a TCS SP-5 confocal microscope (Leica Microsystems, Wetzlar, Germany) equipped with 63 × HCX PL APO oil-immersion optics. Images were captured with a scanning speed of 400 Hz and image resolution of 512 × 512 pixels and then analyzed by Leica Application Suite 2.02.

### *In vivo* angiogenesis

For the *in vivo* studies of exosomes-induced angiogenesis, we used 6–8 week old BALB/c athymic nude mice. Mice (*n* = 5) were subcutaneously injected with 0.5 mL Matrigel (BD Biosciences) containing HUVECs and 20 mg of NPC-derived exosomes or PBS. At day 7, mice were killed, and Matrigel were recovered and stained with hematoxylin and eosin. The vessel area was planimetrically assessed as percentage area per field using ImageJ software (National Institutes of Health).

### *In vitro* angiogenesis

*In vitro* formation of capillary-like structures was studied on HUVECs (5 × 10^4^ cells/well) seeded on Matrigel (BD Biosciences) diluted 1:1 in ice with cold DMEM (Sigma-Aldrich). After cells had attached, the medium was removed and 1 mL medium containing NPC-derived exosomes was added. Experimental results were recorded after 6-hour incubation at 37°C.

### Calculation and statistical analysis

Results from at least three independent experiments are reported as the means ± standard deviation (SD). Statistical analyses were performed using SPSS17.0 software. Survival curves were estimated by Kaplan-Meier analysis and compared by the log-rank test. *χ2* test was used to determine the significance of differences in multiple comparisons. Statistical significance was assessed by 2-tailed Student's *t* test for 2 groups and one-way analysis of variance (ANOVA) for more than 2 groups. *P* < 0.05 was considered statistically significant.
